# Promoting the production of challenging proteins via induced expression in CHO cells and modified cell-free lysates harboring T7 RNA polymerase and mutant eIF2α

**DOI:** 10.1016/j.synbio.2024.03.011

**Published:** 2024-03-27

**Authors:** Jeffrey L. Schloßhauer, Lena Tholen, Alexander Körner, Stefan Kubick, Sofia Chatzopoulou, Anja Hönow, Anne Zemella

**Affiliations:** a Fraunhofer Project Group PZ-Syn of the Fraunhofer Institute for Cell Therapy and Immunology-IZI, Branch Bioanalytics and Bioprocesses-IZI-BB, Am Mühlenberg, Potsdam, Germany; b Fraunhofer Institute for Cell Therapy and Immunology-IZI, Branch Bioanalytics and Bioprocesses-IZI-BB, Am Mühlenberg, Potsdam, Germany; c Faculty of Health Sciences, Joint Faculty of the Brandenburg University of Technology Cottbus –Senftenberg, The Brandenburg Medical School Theodor Fontane, University of Potsdam, Potsdam, Germany; d Institute for Chemistry and Biochemistry, Laboratory of Protein Biochemistry, Freie Universität Berlin, Thielallee 63, 14195, Berlin, Germany; e Institute of Biotechnology, Technische Universität Berlin, Straße des 17. Juni 135, 10623, Berlin, Germany; f New/era/mabs GmbH, August-Bebel-Str. 89, 14482, Potsdam, Germany

**Keywords:** Inducible expression, CHO cells, Cell-free protein synthesis, CRISPR, T7 RNA polymerase, eIF2, *Rosa26*

## Abstract

Chinese hamster ovary (CHO) cells are crucial in biopharmaceutical production due to their scalability and capacity for human-like post-translational modifications. However, toxic proteins and membrane proteins are often difficult-to-express in living cells. Alternatively, cell-free protein synthesis can be employed. This study explores innovative strategies for enhancing the production of challenging proteins through the modification of CHO cells by investigating both, cell-based and cell-free approaches. A major result in our study involves the integration of a mutant eIF2 translation initiation factor and T7 RNA polymerase into CHO cell lysates for cell-free protein synthesis. This resulted in elevated yields, while eliminating the necessity for exogenous additions during cell-free production, thereby substantially enhancing efficiency. Additionally, we explore the potential of the *Rosa26* genomic site for the integration of T7 RNA polymerase and cell-based tetracycline-controlled protein expression. These findings provide promising advancements in bioproduction technologies, offering flexibility to switch between cell-free and cell-based protein production as needed.

## 1 Introduction

Production of biotechnologically relevant proteins can be achieved by expression of the protein of interest in cultured cells or using cell-free reaction conditions. In cell-based approaches, transient as well as stable transfection is often used for recombinant protein production [1,2]. On the other hand, protein synthesis in cell-free systems can be performed by adding DNA templates to the open reaction, which is driven by the presence of an active cell lysate containing components for transcription and protein translation including ribosomes, aminoacyl tRNA synthetases (aaRS), transcription and translation factors, viral RNA polymerase for transcription, substrates and an energy regeneration system [3,4]. The reaction environment can be further manipulated with additives, such as chaperones, mild detergents, labeled amino acids, and cofactors, to tailor protein synthesis as needed. The addition of the chaperones DnaK and GroEL significantly increased the solubility of cell-free produced proteins based on *Escherichia coli* cell lysate, while the addition of various orthogonal aaRS/tRNA pairs and disulfide isomerase to the open system allowed various non-canonical amino acids (ncaa) to be incorporated into proteins and disulfide bridges to be successfully formed [[5], [6], [7], [8]]. To overcome labour-intensive preparation of supplemented purified components, enzymes were introduced into the bacterial genome and overexpressed to generate cell lysates harboring the desired enzymes for straightforward cell-free protein synthesis [9,10]. As an alternative to the commonly used cell-free protein synthesis based on *E. coli*, eukaryotic cell-free systems can be employed for the synthesis of complex and post-translationally modified proteins [11]. It has been shown that diverse membrane proteins can be produced in active form within 3–20 hours in eukaryotic cell-free systems [12,13]. Frequently applied cell lysates for eukaryotic cell-free protein synthesis are based on cultured human cell lines, *Spodoptera frugiperda* 21 (*Sf*21) cells, Chinese hamster ovary (CHO) cells, tobacco cells, wheat germ cells and yeast cells [[14], [15], [16], [17]]. Among these, CHO cells are of critical importance for biopharmaceutical production, as they often serve as host systems for the production of therapeutic proteins [18]. The advantage of CHO cells is their robustness, scalability, and ability to allow post-translational modifications similar to those in human cells [19]. Furthermore, the in-depth analysis of the CHO cell genome offers new possibilities for advancing cell-free protein synthesis based on CHO lysates [20]. The expression of therapeutic proteins can be evaluated rapidly on a small scale in CHO cell-free systems and the identified conditions can be transferred to CHO cell-based production. To facilitate CHO based cell-free production of site-specifically modified membrane proteins, we recently demonstrated that orthogonal *E. coli* tyrosyl-tRNA synthetase can be integrated into CHO cell lysate to modify the pharmaceutically relevant G protein-coupled adenosine receptor A2a in a cell-free reaction [21].

The choice of the appropriate protein production format strongly depends on the individual protein's requirements. While cell-based production can generate large quantities of post-translationally modified proteins, cell-free synthesis can be used to produce toxic proteins and membrane proteins in a significantly reduced time on a small scale [22]. Those proteins can have a negative impact on cell viability and vitality, and may result in apoptosis when constitutively expressed. Alternatively, strict regulation using inducible promoters permits the cell-based expression of difficult-to-produce proteins to be triggered on demand [23]. Thereby, the level of putatively toxic proteins can be repressed to a level that is not harmful to the host organism. This procedure is particularly useful for large scale processes, that aim to first optimize for growth, and later for expression, when the desired cell density is achieved. Furthermore, controlled expression proves beneficial for cell line development, a process that frequently utilizes low cell densities or single cells, rendering them especially susceptible to any additional stress stimuli [24].

There are multiple inducible systems with diverse functionalities to selectively switch on or off desired genes. CHO cell systems frequently use tetracycline and cumate as inducer, as they robustly trigger pronounced protein expression [[25], [26], [27], [28]]. By contrast, more recently developed light-inducible systems allow for spatiotemporal control of protein production, but currently do not provide a strong expression rate when switching on the commonly used blue light [29,30]. To ensure optimal induction of target protein expression, defined positions in the genome are essential for stable transfection of regulatory molecules such as inducible promoters, repressors, and activators, respectively. In the past, *hprt*, *C12orf35*, *Hipp11*, and *Rosa26* have been identified as target sites for stable transfection of expression cassettes because they provide reproducible results and are subject to reduced epigenetic regulation and gene inactivation [[31], [32], [33]].

In the present work, manipulation of cell-free transcription and translation was aimed to simplify the production of complex proteins and to increase expression levels. To this end, regulatory factors were introduced into CHO cells to produce cell lysates for enhanced cell-free protein synthesis. In order to achieve flexible production of toxic proteins and membrane proteins, a tetracycline-inducible system was developed as an alternative to cell-free protein synthesis, which allowed the controlled cell-based expression of difficult-to-express proteins from the *Rosa26* locus.

## 2 Material and methods

### 2.1 Plasmids and template generation

The plasmid pCAG-T7pol (Addgene #59926) for T7 RNA polymerase expression, pSpCas9(BB)-2A-GFP (Addgene #48138) for Cas9 expression and gRNA_cloning vector (Addgene #41824) were obtained from Addgene. The T7 RNA polymerase sequence was extracted from the UniProt database (P00573; RPOL_BPT7). The gRNA C12orf35-T2: 5′-GCC GGG ACT TAA CCA CTC GA-3′ specific for the *C12orf25* locus was designed according to our previous report [21]. The gRNA Rosa26: 5′-TCAAGCGTGAGCATAAAACT-3′ specific for the *Rosa26* locus was obtained from the literature [33]. Gibson assembly was utilized to clone the gRNA sequence into the gRNA_cloning vector (Plasmid #41824 from Addgene) according to the protocol, as previously described [34]. Plasmids based on the piX 3.0 backbone (Biotech Rabbit) are optimal for cell-free protein synthesis due to the presence of a T7 promotor and a T7 terminator as regulatory sequences for transcription. The plasmid piX4.0-NC-Luc containing a cricket paralysis virus (CrPV) internal ribosome entry site (IRES) for translation initiation was used for cell-free synthesis of firefly luciferase [35]. The piX3.0-Nluc without the CrPV IRES and with a nanoluciferase sequence (Promega) was used for the T7 RNA polymerase assay, while a CrPV IRES was cloned into piX3.0-Nluc to produce the plasmid piX3.0-CRPV-Nluc containing both, a CrPV IRES and nanoluciferase for cell-free synthesis. The pIX4.0-Luc plasmid was used to analyze cap-dependent translation initiation, as described previously [36]. Therefore, 0.1 ng/μl pIX4.0-Luc was used as template in a PCR reaction containing 10x ThermoPol Reaction Buffer (New England Biolabs), 14 mU/μl Deep Vent DNA Polymerase (New England Biolabs), 0.2 mM dNTPs, 0.5 μM of T7-Fw primer 5′-ATGATATCTCGAGCGGCCGCTAGCTAATACGACTCACTATAG-3′ and 0.5 μM of PolyA 50-Rv primer 5′-T(50)CAGATCTTGGTTAGTTAG-3′. The following temperature profile was used: 95 °C for 3 min; 30 cycles of 95 °C for 20 s, 51 °C for 20 s, 72 °C for 90 s; 72 °C for 5 min. The PCR product was purified using DNA Clean & Concentrator Kit (Zymo Research) according to the manufacturer's instructions. The pcDNA3.1-eIF2α-S52A containing a human cytomegalovirus (CMV) promoter and a HiBiT tag N-terminally was purchased from GenScript and was utilized for transient transfection of eIF2α-S52A (wildtype sequence of eIF2α extracted from UniProt P05198) in CHO cells. The donor plasmids DV-Tet-GFP and DV-T7RNAPol-Rosa26 for CRISPR/Cas9 based modification of CHO cells were purchased from Biocat. The TRE promoter and rtTA-2a-Puro sequence was extracted from Addgene (Addgene #60495). Homology arms of the donor plasmid eAzFRS, which was described previously [21], were used to substitute homology arms of DV-T7RNAPol-Rosa26 by fusion PCR to generate the new donor sequence DV-T7RNAPol-C12orf35. The donor plasmids contain a CMV promoter for T7 RNA polymerase expression and downstream an EMCV IRES to initiate translation of Blasticidine independently of T7 RNA polymerase. The plasmids PB-Transposase-Sf21 and PB-T7RNAPol-Sf21 were purchased from Biocat. Expression of both PiggyBac Transposase and T7 RNA polymerase is driven by an OpIE-2 promoter, while the PB-T7RNAPol-Sf21 plasmid additionally contain an bleomycin resistance gene driven by an OpIE-1 promoter. PiggyBac 5- and 3-terminal inverted repeats were extracted from the literature [37] and are located at the terminal ends of the donor sequence, enabling PiggyBac Transposase to integrate the donor sequence into the *Sf*21 genome.

### 2.2 Cell lines and cultivation

CHO–K1 cells from the Leibniz Institute DSMZ-German Collection of Microorganisms and Cell Cultures GmbH (DSMZ no: ACC110) were adapted as a suspension culture. CHO cells were grown in serum-free ProCHO5 medium (Lonza) supplemented with 4 mM Ultraglutamine (Lonza) at 37 °C, 5% CO_2_ and 100 rpm in the CO_2_ Multitron incubator (Infors). *Sf*21 suspension cells were cultivated at 27 °C in serum-free Insect-XPRESS medium (Lonza).

### 2.3 Transient transfection and cell lysate preparation

CHO cells were transiently transfected, as described recently [21]. Briefly, 1.5 μg pcDNA3.1-eIF2α-S52A or pCAG-T7pol expression plasmid/10^6^ cells and 2 μg PEI reagent/10^6^ cells were incubated for 4 h at a cell density of 4 × 10^6^ cells/ml at 37 °C, 5% CO_2_ and 80 rpm in the CO_2_ Multitron incubator (Infors). Afterwards, 150 ml fresh culture medium was added to achieve a 200 ml culture at a cell density of 10^6^ cells/ml for two days at 37 °C, 5% CO_2_ and 100 rpm. CHO cells were harvested and washed, as described previously [14]. Cell lysate was prepared by using the lysing matrix A (MP Biomedicals). The wet cell pellet was transferred to a lysing matrix A tube and cells were disrupted for 5 s and 4 m/s in the presence of dry ice in the cooling chamber of the FastPrep-24 Bead-Beating instrument (MP Biomedicals) and cell lysates were isolated as described previously [14].

### 2.4 Stable transfection and isolation of cells

500 ng DNA was diluted in 100 μl Opti-MEM serum-free medium (Gibco) in a ratio of 2:2:1 (linear donor vector: gRNA vector: Cas9 vector). The DNA was delivered into CHO cells by lipid based transfection using 2.5 μL Lipofectamine LTX (Thermo Fisher Scientific) and 0.5 μL Plus reagent (Thermo Fisher Scientific) according to the manufacturer's instructions. Briefly, the mixture was added to 1 × 10^6^ cells/ml in 500 μL serum-free ProCHO5 medium (Lonza) in a 24-well plate after an incubation time of 20 min. Cells were mixed and 15 min centrifugation with 400 × *g* at room temperature was performed according to a previous report [38]. The cells were incubated for two days at 37 °C and 5% CO_2_. Selection pressure was applied by cultivating CHO cells in culture medium in the presence of 10 μg/ml Blasticidine S Hydrochloride (Sigma-Aldrich) for two weeks. Cell lysates for cell-free protein synthesis were prepared, as described above. Cells were isolated by a BD FACS Aria III flow cytometer (Becton Dickinson) and subjected to genotyping PCR, T7 RNA polymerase assays or protein expression was induced by varying tetracycline concentrations, respectively. Stable integration of T7 RNA polymerase into *Sf*21 cells was achieved using the PiggyBac Transposase system by adding pre-diluted 400 ng DNA in a ratio of 1:1 (PB-Transposase-Sf21: PB-T7RNAPol-Sf21) in 20 μl Sf-900 II medium (Gibco) to pre-diluted 2 μl Insect GeneJuice Transfection Reagent (Sigma-Aldrich) in 20 μl Sf-900 II medium (Gibco). After 15 min of incubation, 160 μl Sf-900 II medium (Gibco) was added to the transfection mixture and the final 200 μl were added to 1 × 10^6^ cells/ml in 300 μL Sf-900 II medium (Gibco) in a 24 well plate. After 48 h incubation at 27 °C selection pressure was applied for two weeks using 250 μg/ml Zeocin (Thermo Fisher Scientific). The *Sf*21 clone pool was expanded to a 500 ml culture in shake flasks and cell lysate was prepared for cell-free protein synthesis as described above.

### 2.5 Cell-free protein synthesis

Cell-free protein synthesis was performed using 1.5 ml reaction tubes in 25 μl in the presence of 40% CHO cell lysates, 30 mM HEPES-KOH (pH 7.5, Carl Roth GmbH), 100 mM sodium acetate (Merck), 3.9 mM magnesium acetate (Merck), 150 mM potassium acetate (Merck), 100 μM amino acids (Merck), 250 μM spermidine (Roche), 2.5 mM Dithiothreitol (Life technologies GmbH), 100 μg/ml creatine phosphokinase (Roche), 20 mM creatine phosphate (Roche), 1.75 mM ATP (Roche), 0.3 mM of UTP (Roche), 0.3 mM CTP (Roche), 0.3 mM GTP (Roche), 0.1 mM of the cap analogue m7G (pp)G (Prof. Edward Darzynkiewicz, Warsaw University, Poland) and 10 μM PolyG. For cap-independent cell-free reactions 60 ng/μl plasmid DNA was used, while 20 ng/μl purified PCR product was added to cap-dependent reactions. Moreover, 1 U/μl T7 RNA polymerase (Agilent) was added to the cell-free reaction unless otherwise stated. The cell-free reaction was incubated for 3 h at 30 °C and 600 rpm. Cell-free protein synthesis based on *Sf*21 cell lysates was performed equivalent to CHO based cell-free reactions.

### 2.6 T7 RNA polymerase assay

The activity of T7 RNA polymerase in CHO cells was determined by co-transfecting gWiz-GFP (Genlantis) containing a CMV promoter and piX 3.0-Nluc containing a T7 promoter using Lipofectamine LTX (Thermo Fisher Scientific) in 96-well format according to the manufacturer's instructions. Therefore, 50 ng gWiz-GFP and 50 ng piX3.0-Nluc were combined and added to 0.5 μL Lipofectamine LTX (Thermo Fisher Scientific) and 0.1 μL Plus reagent (Thermo Fisher Scientific) in 10 μl Opti-MEM serum-free medium (Gibco). After 10 min at room temperature 10 μl transfection mixture was directly added to CHO cells and was incubated for two days at 37 °C and 5% CO_2_. Technical duplicates were performed for each independent transfection. The amount of GFP positive cells was determined by the LUNA-FL Dual Fluorescence Cell Counter (Logos Biosystems) according to the manufacturer's instructions. Nanoluciferase activity was analyzed as described below. The detected relative luminescence units were divided by the amount of GFP positive cells. The fold change of samples relative to the CHO clone pool was calculated to compare independent experiments.

### 2.7 Luciferase assays

Firefly luciferase was analyzed by adding 50 μl Luciferase Assay Reagent (Promega) to 5 μl of translation mixture after cell-free protein synthesis. The concentration of active firefly luciferase was determined using a calibration curve. Luminescence was detected using the LB 941 luminometer (Berthold Technologies). Nanoluciferase (Promega) activity was detected using the Nano-Glo Luciferase Assay System (Promega) according to the manufacturer's instructions. Briefly, 100 μl of reagent was added to 100 μl of cells and incubated for 5 min at 300 rpm. For cell-free produced nanoluciferase 5 μl translation mixture after cell-free protein synthesis was mixed with 50 μl reagent and incubated for 3 min. Detection of HiBiT (Promega)-tagged eIF2α-S52A in CHO cells was achieved using the Nano-Glo HiBiT Lytic Detection System (Promega) according to the manufacturer's instructions. Briefly, 100 μl Nano-Glo HiBiT Lytic Reagent containing buffer, substrate and LgBiT for luciferase complex formation was added to 100 μl cell suspension and incubation was performed for 10 min at 300 rpm. The luminescence signal of the HiBiT and nanoluciferase assay was detected by the Multimode Microplate Reader Mithras 2 LB 943 (Berthold Technologies) using an OD2 filter.

### 2.8 Fluorescence microscopy and image analysis

GFP fluorescence of tetracycline induced CHO cells was visualized by the Olympus IX83 inverted microscope combined with the cellSens imaging software (Olympus). The FITC channel was utilized to detect GFP fluorescence. ImageJ v1.54 d software was used to analyze fluorescence images. Therefore, grayscale images were converted to green channel images and the signal intensity was adjusted equally for all images.

### 2.9 Western blot

The cell pellet of 5 ml CHO cell suspension was disrupted 24 h post-transfection by resuspending the pellet in 0.3 ml RIPA lysis buffer (10 mM Tris–HCl pH 8, 140 mM NaCl, 1% Triton X-100, 1 mM EDTA, 0.5 mM EGTA, 0.1% SDS, 0.1% sodium deoxycholate). After incubating cells for 30 min at 4 °C, cells were passed through a syringe tip and cell lysate was isolated by centrifugation at 16,000 × *g* at 4 °C for 20 min. The protein concentration of samples was determined using the Pierce BCA Protein Assay Kit (Thermo Fisher Scientific) and 11.5 μg protein was heated at 70 °C for 10 min in LDS sample buffer (Invitrogen). Samples were separated by denaturing polyacrylamide gel electrophoresis with NuPAGE 10% Bis-Tris Gels (Invitrogen). The protein transfer to a PVDF membrane was performed using the iBlot Dry Blotting System (Invitrogen). The membrane was blocked with 2% bovine serum albumin (BSA) in TBS/T over night at 4 °C. The primary rabbit *anti*-T7 RNA polymerase antibody was diluted 1:1000 in blocking buffer and the membrane was incubated for 3 h under agitation at room temperature. After washing the membrane the 1:2000 diluted secondary anti-rabbit IgG HRP-linked antibody was incubated on the membrane for 1 h. Detection of signals was achieved using the Typhoon TRIO + imager (GE-Healthcare) after washing the membrane and incubating with ECL-detection reagent (GE-Healthcare) for 3 min.

### 2.10 Genotyping PCR

Genomic DNA of stable transfected CHO cells was extracted using the Quick-DNA Miniprep Plus Kit (Zymo Research) according to the manufacturer's instructions. Utilized genotyping primer sequences were designed as followed: one flanking the integration side of the expression cassette inside the *Rosa*26 locus (Rosa26: 5′-GAGGAGGAGATACCCATCTG-3′) and the second one binding in the donor sequence (Tet-GFP: 5′-GGTGCATGACCCGCAAG-3′ and T7RNAPol: 5′-TCCCGACGGATTCCCTGTTT-3′). The 20 μl PCR reaction was composed of 5x Q5 Reaction Buffer (New England Biolabs), 0.02 U/μl Q5 Hot Start High-Fidelity DNA Polymerase (New England Biolabs), 0.5 μM of each primer, 0.2 mM dNTPs and 50 ng genomic DNA. The following temperature profile was used: 98 °C for 2 min; 35 cycles of 98 °C for 10 s, 65 °C for 20 s, 72 °C for 30 s; 72 °C for 2 min. The PCR-products were run on a 1% agarose gel and product size was compared to the Quick-Load 2-Log DNA Ladder (0.1–10.0 kbp, New England Biolabs).

### 2.11 Data and statistical analysis

Statistical analysis was performed using Origin (Pro) software version number 2019 (OriginLab Corporation). Data were presented as mean and standard deviation (SD), when technical replicates were shown. Technical replicates were averaged for independent experiments before the mean and standard error of the mean (SEM) for the independent experiments was calculated. The difference between independent experiments was analyzed by a Student's t-test. Alternatively, the Mann-Whitney *U* test was performed to compare sample means of the T7 RNA polymerase assay, because data were not normally distributed. A p-value <0.05 was considered statistically significant.

## 3 Results

In previous studies, we demonstrated that CHO cells could be modified by transient and stable transfection such that the resulting cell lysates were ideally applicable for straightforward site-specific modification of difficult-to-produce proteins in cell-free reactions [21]. Further improvement of cell-free reaction conditions can be achieved by modifications of the transcriptional and the translational apparatus.

### 3.1 Harnessing CHO cell lysate containing endogenous T7 RNA polymerase for cell-free protein synthesis

One goal of the present study was to examine whether transcription in cell-free protein synthesis based on T7 RNA polymerase can be designed in a more economical and simplified way, as the enzyme represents a cost factor. Moreover, the T7 RNA polymerase is solubilized in a buffer, which contains e.g. ions and glycerol, thus can interfere with cell-free protein synthesis.

**Fig. 1:**
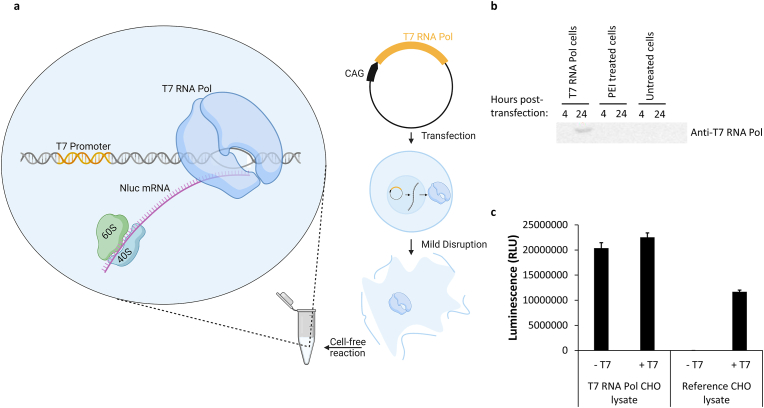
**Cell-free protein synthesis based on transiently transfected CHO cells expressing T7 RNA polymerase.** CHO cells were transiently transfected with a T7 RNA polymerase (T7 RNA Pol) encoding expression plasmid and cell lysate preparation was achieved two days post-transfection. a) Scheme of T7 RNA polymerase expression in cells and action during cell-free reaction. b) Detection of T7 RNA polymerase expression using western blot. The uncropped image can be found in Supplementary Fig. 1 c) Cell-free synthesis of nanoluciferase was carried out with (+T7) and without (-T7) supplemented T7 RNA polymerase using a CHO cell lysate containing endogenous T7 RNA polymerase or CHO lysate without T7 RNA polymerase (reference lysate). Measurements were performed in technical triplicate. Data are shown as mean ± SD.

T7 RNA polymerase plays a critical role in cell-free reactions, as it is used to produce mRNA templates for protein expression by using linear and circular DNA templates. It transcribes DNA into mRNA, which then serves as a blueprint for protein production. It is widely used in various cell-free systems due to its high catalytic rate, stability and precision [39]. Consequently, the present work aimed to investigate whether viral T7 RNA polymerase can be integrated into CHO cells prior to cell lysate preparation. Subsequently we analyzed if cell lysates are suitable for cell-free protein synthesis without further addition of RNA polymerase (Fig. 1a). Therefore, CHO cells were transiently transfected with T7 RNA polymerase encoding plasmid DNA and cell lysate was generated two days post-transfection. T7 RNA polymerase expression was observed in transfected cells 24 h post-transfection by western blotting (Fig. 1b). As expected, T7 RNA polymerase could not be detected in untreated cells and cells only treated with PEI reagent.

The new cell lysate was utilized to examine T7 RNA polymerase activity in cell-free protein synthesis using a nanoluciferase (Nluc) template containing a T7 Promoter to initiate transcription and a CrPV IRES to initiate protein translation. In the absence of supplemented T7 RNA polymerase ∼90% of the luminescence signal of the sample supplemented with the polymerase could be obtained, when using the novel cell lysate with endogenous enzyme (Fig. 1c). In contrast, cell-free protein synthesis based on non-transfected CHO cells only produced a luminescence signal after the addition of purified T7 RNA polymerase to the reaction.

### 3.2 Boosting cell-free protein synthesis by influencing cap-dependent protein translation initiation

**Fig. 2:**
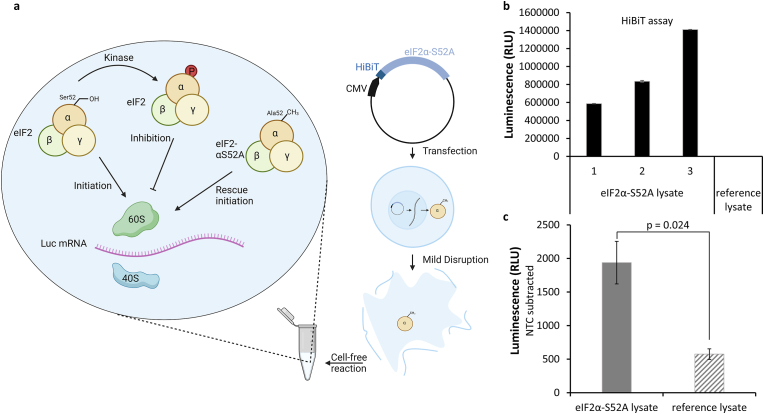
**Cell-free protein synthesis based on transiently transfected CHO cells expressing eIF2α-S52A.** CHO cells were transiently transfected with an eIF2α-S52A expression plasmid, containing a HiBiT tag at the N-terminus, and cell lysate preparation was achieved two days post-transfection. a) Scheme of eIF2α-S52A expression in cells and action of eIF2α-S52A during cell-free protein synthesis. b) Detection of eIF2α-S52A expression using the HiBiT assay. Three independent transfections and cell lysate preparations (sample 1–3) were performed and data is expressed as technical duplicate. c) Cell-free synthesis of firefly luciferase utilizing CHO cell lysate containing endogenous eIF2α-S52A. A cell-free reaction without luciferase template (No-template control, NTC) was subtracted from sample values for background subtraction. Three independent transfections were performed to produce eIF2α-S52A CHO lysates (grey), while six independent cell lysate preparations were performed from non-transfected CHO cells referred as to reference lysate (diagonal lines). Data are shown as mean ± SEM. Statistical significance was observed by a *t*-test.

The initiation of protein translation is the rate-determining step and has a crucial impact on the efficiency of cell-free protein synthesis [40]. Modification of the cap-dependent initiation allows precise control over protein expression levels. By varying the 5′ cap structure or the initiation factors, the expression level can be optimally controlled [41,42]. In contrast, CrPV IRES-dependent initiation, which is often used in cell-free reactions, is more difficult to control or modify because it is independent of the cap structure and initiation factors [14,43,44]. A significant regulator of translational initiation is the initiation factor eIF2α. Phosphorylation of eIF2α at Ser 52 by eIF2α kinases, often triggered by various stress responses, causes decreased eIF2 charging to GTP and thus leads to a reduction in overall protein synthesis [45,46]. Previously it was shown that mutating the phosphorylatable serine to alanine resulted in increased protein expression in CHO cells [47]. Based on this, it was intended to test whether transfection of the eIF2α-S52A mutant would lead to increased protein synthesis rates in cell-free protein synthesis (Fig. 2a). First, a N-terminal HiBiT tag, was utilized to detect successful HiBiT-eIF2α-S52A expression based on a split nanoluciferase assay, which is highly sensitive due to the strong luminescence activity of nanoluciferase. Indeed, eIF2α-S52A expression could be observed, while no luminescence signal could be obtained for untreated cells (Fig. 2b). Three independent transfections and cell lysate preparations were carried out to reproduce these findings. Subsequently, firefly luciferase was synthesized cap-dependently in eIF2α-S52A modified and unmodified cell lysates in cell-free reactions. A significant increase in protein synthesis rate was inferred based on a 3.4-fold higher luminescence signal, in lysates harboring eIF2α-S52A when compared to unmodified lysates (Fig. 2c).

### 3.3 Improvement of cell-free protein synthesis by integrating the T7 RNA polymerase in genomic safe harbor sites

**Fig. 3:**
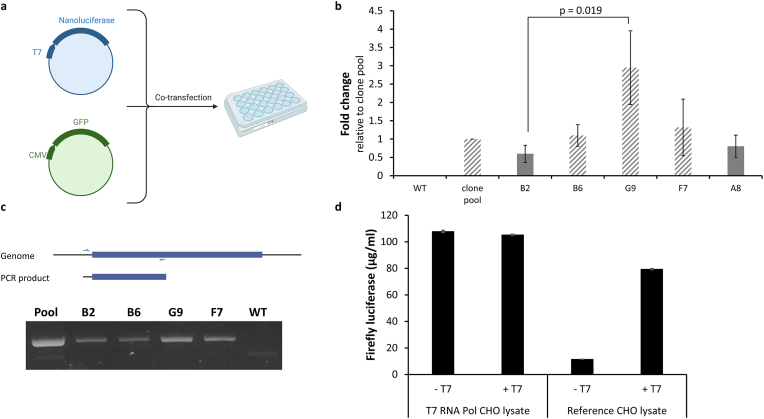
**Generation of stably transfected CHO cells expressing T7 RNA polymerase.** CHO cells were stably transfected by CRISPR/Cas9 to integrate a T7 RNA polymerase expression cassette into *Rosa26* in the CHO genome. a) Scheme of the T7 RNA polymerase assay used for the analysis of T7 RNA polymerase expressing CHO cells. A nanoluciferase plasmid driven by a T7 promoter and an expression plasmid containing a CMV promoter upstream of a GFP gene was utilized to evaluate T7 RNA polymerase expressing CHO cells. b) T7 RNA polymerase assay. The fold change is relative to the clone pool from which the cell lines were selected from. Independent transfections (grey: n = 4; diagonal lines: n = 6) were carried out. Data are shown as mean ± SEM. A Mann-Whitney *U* test was used to detect statistical significance. c) Genotyping PCR was performed using a primer pair binding inside and outside of the donor template. The resulting PCR products were analyzed by agarose gel electrophoresis. The uncropped image can be found in Supplementary Fig. 3) d)Cell-free synthesis of firefly luciferase was carried out with (+T7) and without (-T7) supplemented T7 RNA polymerase using a CHO cell lysate containing endogenous T7 RNA polymerase or CHO lysate without T7 RNA polymerase (reference lysate). Measurements were performed in technical duplicate. Data are shown as mean ± SD.

After demonstrating enhanced cell-free protein synthesis by manipulating transcription and translation using T7 RNA polymerase or mutant eIF2α, we next intended to perform stable transfection of CHO cells to generate modified cell lysates. Stable transfection of T7 RNA polymerase should demonstrate that a suitable locus is present in the CHO genome for future gene integrations. For this purpose, the homologous *Rosa26* locus was compared to the *C12orf35* locus. In a previous study, we used *C12orf35* as a target site and showed that it is capable of expressing the orthogonal *E. coli* tyrosyl-tRNA synthetase, resulting in translationally active cell lysates that can be used for orthogonal cell-free protein synthesis [21]. Gaidukov et al. demonstrated that the *Rosa26* locus, which is commonly used for transgene site-specific integration in human and mouse cell lines, is also present in homologous form in the CHO genome [33]. Loci were analyzed using a T7 RNA polymerase assay based on a dual reporter system (Fig. 3a). A T7 promoter upstream of the Nluc gene was utilized to evaluate transcriptional efficiency corresponding to the luminescent signal. At the same time, another plasmid was co-transfected, which contains a CMV promoter upstream of a GFP gene for constitutive expression. The luminescence signal was normalized to the GFP positive cells to compensate for differences such as cell number and transfection efficiency. The clone pools enriched by selection pressure were transfected and analyzed in two independent experiments. A 88 ± 29 fold higher luminescence signal was obtained with the *Rosa26* clone pool compared to the *C12orf35* clone pool. After isolation of clonal cell lines from the promising *Rosa26* pool, again the T7 RNA polymerase assay was performed to evaluate the outgrown T7 RNA polymerase expressing cell lines. The CHO clone G9 achieved the highest T7 RNA polymerase activity among the tested clones and significantly higher activity than CHO clone B2 (Fig. 3b). After further expansion of cell lines, genotyping PCR was performed by using a primer which binds inside the T7 RNA polymerase expression cassette and a primer which binds outside of the expression cassette to ensure gene integration at the *Rosa26* genomic site (Fig. 3c). CHO clone A8 showed decreased cell viability and was not examined further. The T7 RNA polymerase expression cassette seems to be integrated at the desired *Rosa26* locus, indicated by the PCR product. As a result, CHO G9 clone was the ideal candidate to produce cell lysate for cell-free protein production. Consequently, it was examined whether the cell lysate containing the T7 RNA polymerase can be used for cell-free protein synthesis. The firefly luciferase plasmid containing a T7 promoter upstream of the coding sequence, was added to the cell-free reaction to evaluate the new CHO lysate. Cell-free reactions with and without the addition of commercial T7 RNA polymerase were performed by using the novel T7 RNA polymerase harboring lysate and a reference lysate, which was derived from non-modified CHO cells. As expected, no signal was detected without the addition of T7 RNA polymerase to the cell-free reaction based on unmodified CHO cells (Fig. 3d). In contrast, with the newly developed cell lysate, a comparable luminescence signal was obtained without the addition of T7 RNA polymerase compared to the cell-free reaction with further addition of commercial T7 RNA polymerase.

We transferred the approach to *Sf*21 cell-free reactions by a PiggyBac transposase system to stably transfect T7 RNA polymerase into *Sf*21 cells. Although, T7 RNA polymerase activity was also detected during cell-free protein synthesis, further enzyme addition to the cell-free reaction is of distinct advantage (Supplementary Fig. 2).

### 3.4 Tetracycline-driven gene expression at the *Rosa26* locus

Once established that the *Rosa26* locus is well adapted for integration of genes for cell-free protein synthesis without affecting the sensitive translational active lysate, we further investigated whether this locus can be utilized to control gene expression in cells by addition of an induction agent. Consequently, we decided to develop a tetracycline inducible system at the *Rosa26* genomic site to flexibly switch between cell-free and cell-based production depending on the application.

**Fig. 4:**
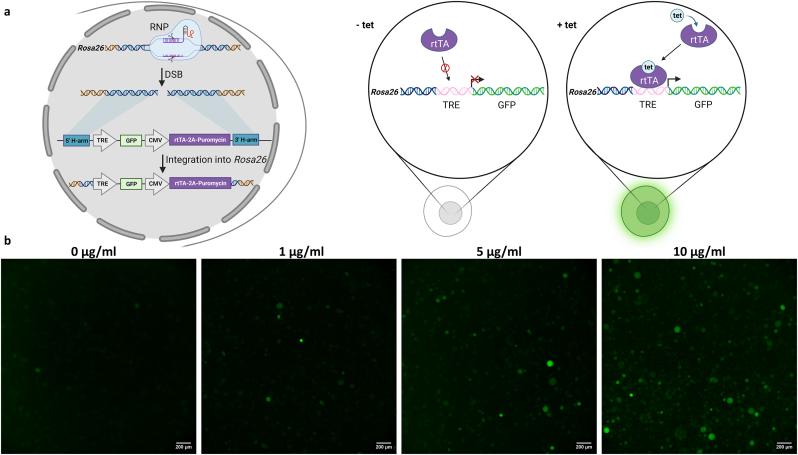
**Tetracycline-based induction of GFP expression at *Rosa26* in CHO cells.** CHO cells were stably transfected by CRISPR/Cas9 with an all-in-one donor template to integrate a GFP expression cassette controlled by a tetracycline-responsive element (TRE) and an expression cassette containing the reverse tetracycline-controlled transactivator (rtTA) driven by a CMV promoter into *Rosa26* in the CHO genome. a) Scheme of CRISPR/Cas9 based modification of CHO cells to create cells capable of tetracycline (tet) -based induction of GFP expression in the presence (+tet) or absence (-tet) of tetracycline. b) A clonal cell line was isolated and incubated in the absence (0 μg/ml) and presence of increasing tet concentrations (1–10 μg/ml). CHO cells were analyzed by fluorescence microscopy two days after tet addition and images represent the fluorescence detection by using the FITC channel.

For this purpose, we designed an all-in-one donor vector containing the tetracycline-inducible tetracycline-response element (TRE) coupled to a CMV promoter upstream of a GFP gene (Fig. 4a). Downstream of the inducible expression site, the reverse tetracycline-controlled transactivator (rtTA) was under the control of a constitutive CMV promoter. Upon addition of tetracycline, the rtTA can bind to the TRE element and start transcription. The rtTA was fused to a puromycin resistance gene via a self-cleavage peptide site to allow enrichment and isolation of clonal cells. The large construct of 4554 bp (without plasmid backbone) was stably transfected into the *Rosa26* locus using CRISPR/Cas9. Correct integration was confirmed by genotyping PCR (Supplementary Fig. 3). Without the addition of tetracycline to the culture medium only weak fluorescence could be detected by microscopy (Fig. 4b). As anticipated, elevating tetracycline levels in the medium resulted in increased fluorescence signals. Thus, it was demonstrated for the first time that an inducible system could be successfully used at the *Rosa26* locus in the CHO genome.

## 4 Discussion

Eukaryotic cell-free protein synthesis is an ideal reaction format to rapidly produce complex proteins that are difficult to manufacture. However, protein synthesis under cell-free conditions is costly, often limiting protein production in larger quantities. In the present study, we demonstrated that cell-free protein synthesis based on CHO cell lysates can be more efficient and cost-effective by modifying the host cells. The potential of enhancing cell-free cap-dependent translation initiation was recently demonstrated by overexpression of GADD34 and K3L in human cells [48]. It was shown that increased levels of the truncated protein phosphatase GADD34 and the vaccinia virus protein K3L prior to cell disruption resulted in decreased eIF2α phosphorylation and increased cell-free protein production based on human cell lysate. We have previously shown that eIF2α phosphorylation is significantly increased in the absence of the specific kinase PERK inhibitor C38 in the cell-free reaction [36], thus the presence of eIF2α-S52A in the cell lysate can compensate for translation initiation in cell-free reactions. Underhill et al. transiently transfected CHO cells with the mutant eIF2α to prevent phosphorylation and could achieve a 3-fold increase of reporter gene activity compared to control cells, which is in accordance with our findings, based on the mutant eIF2α cell lysate [47].

Frequently, the CrPV IRES is utilized for translation initiation in eukaryotic cell-free systems because it is independent of the cap structure and translation initiation factors [49,50]. Although the Encephalomyocarditis virus (EMCV) IRES is commonly used in mammalian cell lines, its effectiveness in cell-free systems was poor [14,51,52]. The EMCV IRES operates independently of the cap structure, but unlike CrPV IRES, initiation factors such as eIF2 are required [53]. Therefore, using the novel mutant eIF2α cell lysate, translational initiation rates, based on diverse IRES, could potentially be increased. T7 RNA polymerase is a significant cost factor of cell-free protein synthesis, hence *E.coli* based cell-free protein synthesis systems often utilize extracts based on host cells, which have the T7 RNA polymerase integrated into the genome [54]. Here, we were able to establish an eukaryotic cell-free production system that does not require the exogenous addition of T7 RNA polymerase. While lysates based on the expression of orthogonal *E. coli* tyrosyl-tRNA synthetase at the *C12orf35* locus could be successfully used, expression of T7 RNA polymerase at the same target site was not effective. This could be due to the larger coding sequence of the T7 RNA polymerase (∼2.65 kbp) to be inserted in contrast to the orthogonal aaRS (∼1.27 kbp). Histone deacetylation or DNA methylation of the gene are known to decrease recombinant protein production during ongoing cultivation [55], thus epigenetic silencing could result in low T7 RNA polymerase expression at *C12orf35*.

While the *Rosa26* locus has become standard as target site in human and mouse cell lines for many years, it has been shown that the homologous *Rosa26* in the CHO genome can be used to exchange expression cassettes for constitutive expression [33,56,57]. In the present work, *Rosa26* was found to be a suitable target site that allows for the incorporation of large expression cassettes, such as the complete regulatory units required for induced expression in CHO cells. Thus, the *Rosa26* target site provides a safe, predictable location in the CHO genome where foreign DNA can be introduced for inducible protein expression without disrupting normal gene function. Thus, expression of the desired proteins can be regulated under specific conditions without undesirable effects on other genetic processes.

It is often observed that the tetracycline-based system may have low basal activity in the absence of tetracycline or the derivative doxycycline [[58], [59], [60]]. We found minimal basal activity of the established system in the absence of tetracycline, but at the same time a significant increase in expression with increasing tetracycline concentration. Alternatively, other inducible systems that exhibit lower basal activity, such as light-inducible systems or riboswitches, could be used in the future, depending on the desired level of expression [29,61]. Furthermore, it would be interesting to use the established system for the induction of toxic proteins to compare them with cell-free protein synthesis or to produce initially cell-free characterized toxins and membrane proteins in a cell-based manner. Moreover, targeted S52A modification of endogenous eIF2α by CRISPR/Cas prior to cell disruption could shift the balance between phosphorylated and non-phosphorylated eIF2α to enhance translation initiation without stressing cells by permanently increasing eIF2α activity. Combined recombinant expression of orthogonal aaRS, T7 RNA polymerase and eIF2α-S52A can be subject of gene silencing and a burden for living cells, when inserted in undefined loci. In the future, combined stable transfection of desired proteins could be conceivable using the *Rosa26* locus to improve cell-free protein synthesis.

## 5 Conclusion

In this study, we increased the protein synthesis rate by modification of translation initiation using a eIF2α-S52A modified CHO cell lysate. Additionally, the integration of T7 RNA polymerase into the CHO cell lysate by transient and stable transfection decreased costs of cell-free protein synthesis. The utilization of the *Rosa26* locus enabled us to develop an effective tetracycline-controlled expression system. Anticipating the future, we are confident that the combination of the cost-effective cell-free and the cell-based *Rosa26* inducible system, provide flexible production of difficult-to-express proteins for diverse applications in biotechnology.

## Funding

This work was supported by the European Regional Development Fund (10.13039/501100008530EFRE) and the German Ministry of Education and Research (10.13039/501100002347BMBF 031B0831C).

## CRediT authorship contribution statement

**Jeffrey L. Schloßhauer:** Conceptualization, Methodology, Formal analysis, Visualization, Investigation, Writing – original draft. **Lena Tholen:** Methodology, Supervision. **Alexander Körner:** Methodology, Writing – review & editing. **Stefan Kubick:** Supervision, Writing – review & editing. **Sofia Chatzopoulou:** Investigation. **Anja Hönow:** Resources. **Anne Zemella:** Supervision, Writing – review & editing.

## Declaration of competing interest

The authors declare that they have no known competing financial interests or personal relationships that could have appeared to influence the work reported in this paper.
