# Supplementary material for: Promoting the production of challenging proteins via induced expression in CHO cells and modified cell-free lysates harboring T7 RNA polymerase and mutant eIF2α
Source: Synth Syst Biotechnol. 2024 Mar 27;9(3):416–24. doi: 10.1016/j.synbio.2024.03.011 (PMC11004649; doi:10.1016/j.synbio.2024.03.011)
Supplement: Multimedia component 1 [file mmc1.pdf]

Supplementary Information

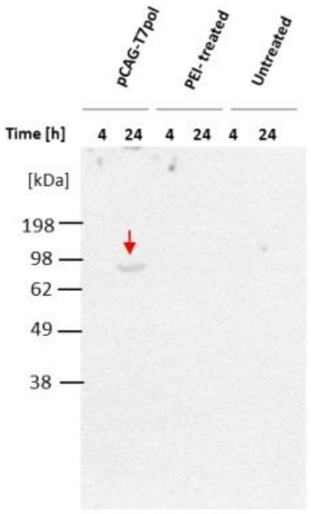

Supplementary Figure 1: Western blot. Detection of T7 RNA polymerase expression using western blot. Displayed is the uncropped image of Figure 1b. A SeeBlue Plus2 Pre-Stained Standard (Life technologies) was used. The red arrows indicates the protein band of T7 RNA polymerase.

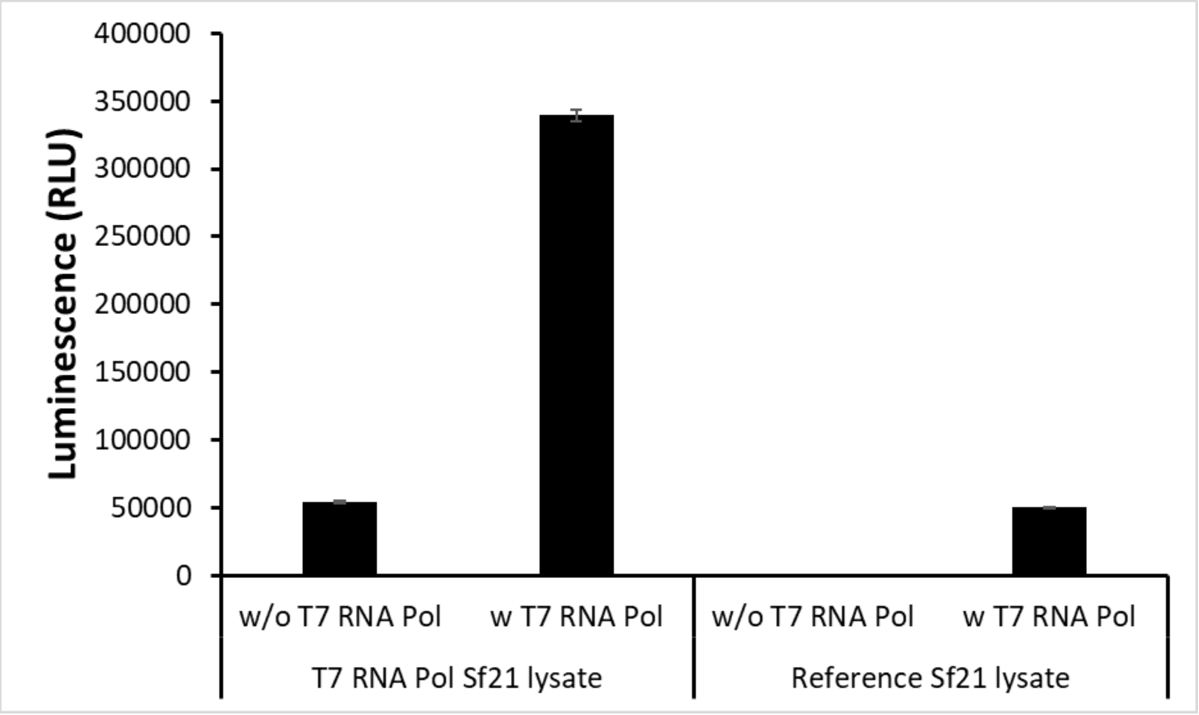

Supplementary Figure 2: Cell-free synthesis of luciferase based on *Sf*21 cell lysates containing endogenous T7 RNA polymerase. *Sf*21 cells were stable transfected using PiggyBac Transposase. Positively stable transfected cells were enriched in the presence of the selection marker Zeocin. Cell-free synthesis of the Luciferase was carried out with (w) and without (w/o) supplemented T7 RNA polymerase using a *Sf*21 cell lysate containing endogenous T7 RNA polymerase or *Sf*21 lysate without T7 RNA polymerase (reference lysate). Measurements were performed in technical duplicate. Data are shown as mean ± SD.

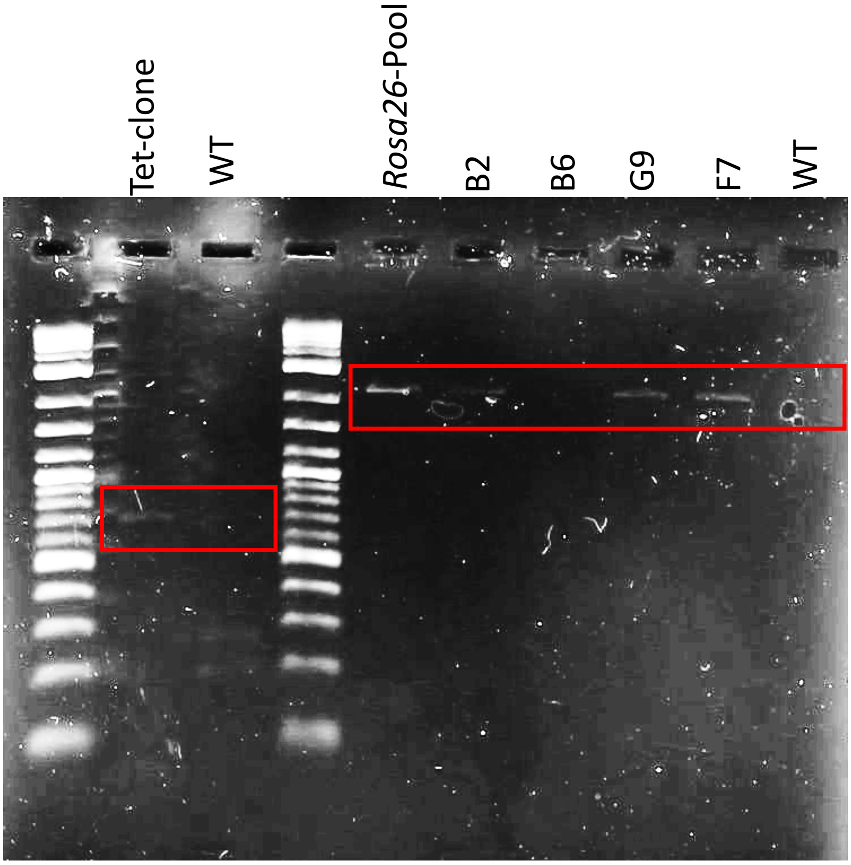

Supplementary Figure 3: Genotyping PCR. Genotyping of the tetracycline-inducible CHO cell line and the T7 RNA polymerase expressing CHO cell lines was performed by PCR using a primer pair binding inside and outside of the donor template. The resulting PCR products were visualized by agarose gel electrophoresis. Red boxes indicate the expected PCR products. Lane 1 & 4: Quick-Load 2-Log DNA Ladder 0.1–10.0 kb (New England Biolabs). Lane 2: tetracycline-inducible CHO cell line, Lane 3 & 10: Wildtype CHO cells (WT), Lane 5: *Rosa26* clone pool expressing T7 RNA polymerase, Lane 6-9: CHO clones expressing T7 RNA polymerase. Genotyping of T7 RNA polymerase expressing cells was repeated and is presented in Supplementary Figure 4.

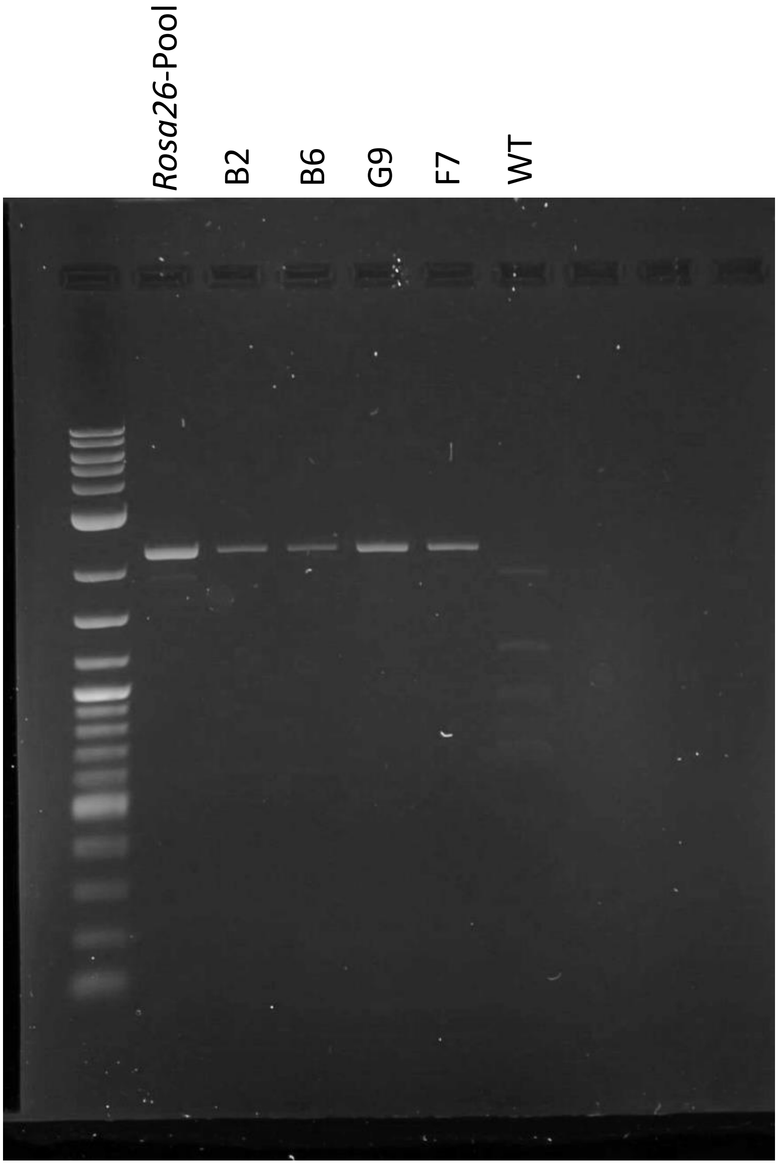

Supplementary Figure 4: Repeated genotyping PCR of T7 RNA polymerase expressing cells. Genotyping was repeated due to the absence of PCR-products for clone B6. Lane 1: Quick-Load 2-Log DNA Ladder 0.1–10.0 kb (New England Biolabs), Lane 2: *Rosa26* clone pool expressing T7 RNA polymerase, Lane 3-6: CHO clones expressing T7 RNA polymerase, Lane 7: Wildtype CHO cells (WT).
